# Defining the structure of the NF-ĸB pathway in human immune cells using quantitative proteomic data

**DOI:** 10.1016/j.cellsig.2021.110154

**Published:** 2021-12

**Authors:** Fatma O. Kok, Haoying Wang, Patricia Riedlova, Carl S. Goodyear, Ruaidhrí J. Carmody

**Affiliations:** Centre for Immunobiology, Institute of Infection, Immunity & Inflammation, College of Medicine, Veterinary and Life Sciences, University of Glasgow, Glasgow G12 8TA, United Kingdom.

## Abstract

The NF-ĸB transcription factor is a critical regulator of immune homeostasis and inflammatory responses and is a critical factor in the pathogenesis of inflammatory disease. The pathways to NF-ĸB activation are paradigms for signal-induced ubiquitination and proteasomal degradation, control of transcription factor function by subcellular localisation, and the control of gene transcription and physiological processes by signal transduction mechanisms. Despite the importance of NF-ĸB in disease, the NF-ĸB pathway remains unexploited for the treatment of inflammatory disease. Our understanding of NF-ĸB comes mostly from studies of transgenic mice and cell lines where components of the pathway have been deleted or over expressed. Recent advances in quantitative proteomics offer new opportunities to understand the NF-ĸB pathway using the absolute abundance of individual pathway components. We have analysed available quantitative proteomic datasets to establish the structure of the NF-ĸB pathway in human immune cells under both steady state and activated conditions. This reveals a conserved NF-κB pathway structure across different immune cell lineages and identifies important differences to the current model of the NF-ĸB pathway. These include the findings that the IKK complex in most cells is likely to consist predominantly of IKKβ homodimers, that the relative abundancies of IκB proteins show strong cell type variation, and that the components of the non-canonical NF-ĸB pathway are significantly increased in activated immune cells. These findings challenge aspects of our current view of the NF-κB pathway and identify outstanding questions important for defining the role of key components in regulating inflammation and immunity.

## Highlights

• Quantitative proteomic datasets offer new insights into the NF-κB pathway.
• The structure of the NF-κB pathway is highly conserved in human immune cells.
• The IKK complex is likely composed mainly of IKKβ homodimers.
• The relative abundancies of IκBα, −β and -ε show strong cell type variation.
• Components of the non-canonical NF-ĸB pathway are greatly increased by activation.

## 1 Introduction

Since its discovery over 30 years ago, NF-κB has served as a model for inducible transcription factors, signal-directed ubiquitination, and the impact of signal transduction pathways on gene expression and physiological processes [1]. The NF-ĸB transcription factor is a master regulator of inflammation and immunity, and a key factor in the pathology of diseases responsible for the majority of deaths worldwide [2]. As such, NF-κB and the pathways that lead to its activation are important targets that have yet to be harnessed for therapeutic benefit.

NF-κB is in fact a family of transcription factors composed of the p65 (RelA), c-Rel, RelB, p50 and p52 subunits that form both homodimers and heterodimers [3]. The p50 and p52 subunits share two key properties that distinguish them from other subunits; the absence of a transactivation domain and their formation through the limited proteasomal processing of the precursor proteins p105 (NFKB1) and p100 (NFKB2) which generate p50 and p52 respectively [3]. In the steady state, NF-κB dimers are sequestered in the cytoplasm through interaction with the IκB family of proteins that include IκBα, IκBβ and IκBε [4]. Also included in this family of NF-κB inhibitory proteins are the p105 and p100 precursor proteins which sequester NF-κB dimers in via a C terminal ankyrin repeat domain homologous to the central ankyrin repeat domains found in IκBα, IκBβ and IκBε [4].

The IKK complex is required for NF-κB activation in response to the majority of immunoreceptors in what is termed the classical NF-κB pathway [3]. The IKK complex is composed of the homologous IKKα and IKKβ kinases bound to the scaffold protein NEMO [5]. Activation of NF-κB follows activation of the IKK complex which phosphorylates IκB proteins, triggering their polyubiquitination and subsequent proteasomal degradation, liberating NF-κB dimers which then translocate to the nucleus [3]. A second, evolutionarily conserved pathway for the activation of NF-κB is triggered in response to a limited number of TNF receptor superfamily members including BAFF receptor, lymphotoxin β receptor, RANK and CD40 [6]. This alternative pathway requires IKKα, but not NEMO nor IKKβ, which phosphorylates p100 leading to its polyubiquitination and subsequent proteasomal degradation. These events lead to the nuclear translocation of NF-κB dimers composed of the RelB and p52 subunits [7]. A negative feedback loop involving the NF-κB-dependent induction of IκB gene expression is the predominant mechanism for terminating NF-κB activity. Newly synthesised IκB proteins translocate to the nucleus where they bind NF-κB dimers and mediate their export from the nucleus. Importantly, the degradation and stimulus-induced expression profile of each IκB subunit appears distinct and may be linked to a specific role for each protein in regulating inflammation. The rapid degradation and resynthesis of IκBα allows for the rapid inhibition of NF-κB activity while the slower kinetics of IκBβ and IκBε degradation may act to dampen long-term oscillations of the NF-κB response [4].

Post-activation, NF-κB is subject to a complex network of post translational modifications, pre-dominantly phosphorylation and ubiquitination, which serve to regulate DNA binding affinity, transactivation activity and protein stability [8], [9]. Phosphorylation of NF-κB subunits occurs at numerous sites and can regulate transcription in a gene selective manner [8], while NF-κB ubiquitination is an important mechanism for terminating transcriptional activity [9]. Activated NF-κB dimers are also regulated by atypical members of the IκB family of proteins which include BCL-3, IκBζ, IκBNS and IκBη. These proteins are predominantly nuclear in localisation and are not degraded following IKK activation [4]. Rather, they serve to modulate the function of NF-κB dimers in the nucleus by enhancing or inhibiting NF-κB transcriptional activity. Similar to the typical IκB proteins the atypical IκB proteins show selectivity in binding to specific NF-κB dimers [4].

The importance of NF-κB in physiological and pathological process has been largely informed by studies of transgenic mice deficient in core components of the NF-κB pathway [10]. The accepted model of the NF-κB pathway is based on biochemical and molecular biology studies often involving overexpression of factors in cell lines or analysis of transformed mouse embryonic fibroblasts derived from transgenic mice. Recent advances in quantitative proteomics present new opportunities to define the structure and composition of the NF-κB pathway.

Here, we analyse publicly available quantitative proteomic data to generate a model of the NF-κB pathway based on the stoichiometry and abundance of individual components in primary human immune cells [11]. This analysis demonstrates a remarkable conservation of the structure and abundance of the NF-κB pathway across different immune cell lineages. It suggests that the composition of the IKK complex in most cells is likely to consist of IKKβ homodimers, and reveals an unexpected variation in the relative abundancies of IκBα, IκBβ and IκBε in different cell types, indicating cell type specific roles. In most cell types, stimulation results in a significant increase in the levels of the RelB and p100 subunits suggesting that the non-canonical pathway plays an important role in activated immune cells. These findings challenge aspects of our current view of the NF-κB pathway and identify outstanding questions important for defining the role of key components in regulating inflammation and immunity.

## 2 Materials and methods

### 2.1 Datasets and analysis

Proteomic data was sourced from the published studies in references [11], [12], [13], [14]. Networks were generated using Cytoscape. Hierarchical clustering and similarity matrix generation was performed using Morpheus (https://software.broadinstitute.org/morpheus).

### 2.2 CD14^+^ monocyte isolation and stimulation

Peripheral blood mononuclear cells (PBMCs) were isolated from fresh blood of healthy donors by density gradient. Blood was diluted 1:1 in sterile phosphate-buffered saline (PBS; Gibco, UK), carefully applied to 3 ml of Ficoll-Paque Plus (Cytiva, UK) and layered by centrifuging at 400*g* for 30 min with no brakes. The top layer (plasma) was discarded and the layer beneath containing PBMCs was collected. PBMCs were washed three times in PBS and centrifuged at 300*g* for 10 min between the washes. CD14^+^ monocytes were isolated from PBMCs with EasySep Human CD14 Positive Selection kit according to the manufacturer's instructions (Stemcell Technologies, UK). The isolated monocytes were subsequently resuspended at 1 × 10^6^ cells/ml in complete Alpha Minimum Essential Medium (α-MEM; Gibco, UK) supplemented with 10% fetal bovine serum (FBS), 1% penicillin/streptomycin and 1% l-glutamine. Cells were stimulated with 100 ng/ml LPS (*Escherichia coli* 055:B5, Sigma, UK) for 16 h prior to analysis by western blotting.

### 2.3 Western blotting

Whole cell lysates were prepared from cells previously washed twice in ice-cold PBS and suspended in RIPA buffer containing 50 mM Tris-HCl pH 7.4, 1% NP-40, 0.25% deoxycholate, 150 mM NaCl, 1 mM EDTA, supplemented with 1 mM PMSF, 1 mM NaF, 1 mM Na3VO4, 2 μg/ml aprotinin, 1 μg/ml pepstatin and 1 μg/ml leupeptin. Lysates were resolved using Tris-Glycine SDS PAGE, transferred to nitrocellulose membranes and immunoblotted with specific antibodies. Anti-p105/p50 (HPA027305) and anti p100/p52 (HPA008422) were obtained from Atlas Antibodies. Anti-cRel (sc-71) and anti-RelB (sc-226) were obtained from Santa Cruz Biotechnology. Anti-p65 (A301-824A) was obtained from Bethyl Laboratories. Anti-α-tubulin antibody (T6074) was purchased from Sigma. Western blots were imaged using a digital chemiluminescence scanner (LiCor).

## 3 Results

**Fig. 1:**
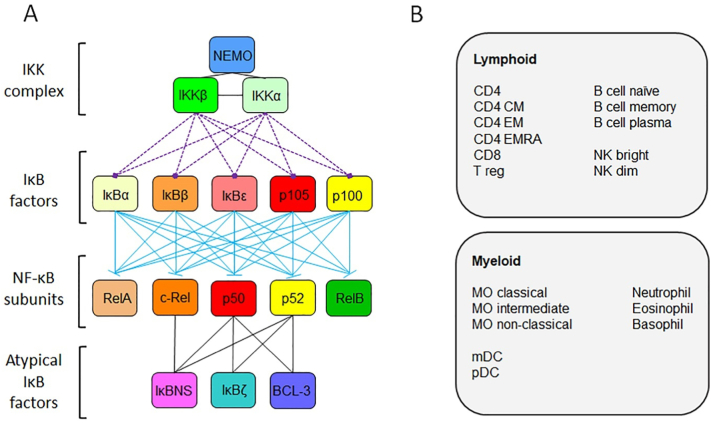
**The NF-κB pathway.** Schematic overview of the NF-ĸB pathway and its component factors **(A)** and **(B)** the proteomic data from human primary cell types from [11] analysed. CD4, CD4^+^ T cells; CD4 CM, CD4^+^ central memory T cells; CD4 EM, CD4+ effector memory T cells; CD4 EMRA, CD4^+^CD45^+^ effector memory T cells; CD8, CD8^+^ T cells; T reg, regulatory T cells; NK bright, natural killer cells CD56^high^; NK dim, natural killer cells CD56^low^; MO classical, monocytes CD14^+^CD16^−^; MO intermediate, monocytes CD14^+^CD16^low^; MO non-classical, monocytes CD14^+^CD16^+^; mDC, myeloid dendritic cells; pDCs, plasmacytoid dendritic cells. Interactions are indicated by black lines; phosphorylation by dashed purple lines; and inhibition by blue lines. (For interpretation of the references to colour in this figure legend, the reader is referred to the web version of this article.)

To determine the structure of the NF-κB pathway in human immune cells we analysed the quantitative proteomic dataset recently generated by Reickman and colleagues [11]. We defined the NF-κB pathway as the IKK complex (NEMO, IKKα and IKKβ), the IκB factors (IκBα, IκBβ, IκBε, p105 and p100), the NF-κB subunits (RelA/p65, c-Rel, p50, p52 and RelB) and the atypical IκB factors (IκBNS, IκBζ and BCL-3) (Fig. 1A). Data on these proteins from lymphoid and myeloid cells at the steady state and following activation were used for the analysis carried out here (Fig. 1B).

### 3.1 Composition of the IKK complex

**Fig. 2:**
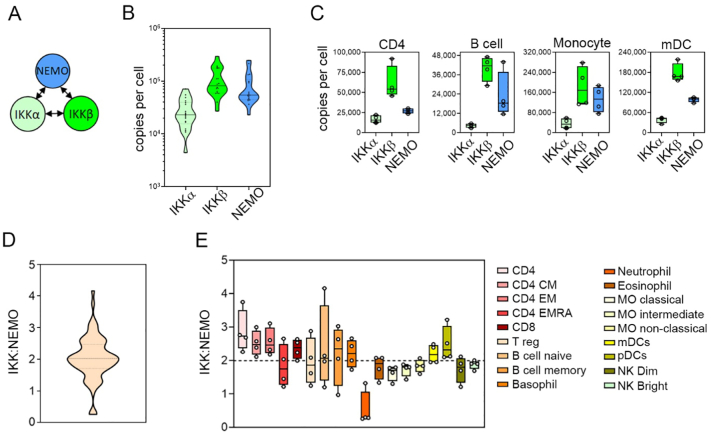
**Stoichiometry of the IKK complex in different human primary immune cell types at the steady state. (A)** The IKK complex is formed from the IKKα and IKKβ kinases, and the NEMO scaffold protein. **(B)** The steady state copy number per cell of IKKα, IKKβ and NEMO for all cell types. The mean value of replicate samples for each cell type is presented. Violin plot shows upper and lower quartiles (dotted lines), and the median (solid line). **(C)** Copy number per cell at the steady state of IKKα, IKKβ and NEMO for CD4^+^ T cells, naïve B cells, monocytes (classical monocytes) and myeloid DCs (mDC). Individual replicate values are shown, mean value is indicated by solid line, boxes extend from the upper to lower quartile and whiskers indicate minimum and maximum values. **(D)** Average ratios of IKKα and IKKβ to NEMO at the steady state for all cell types. Violin plot shows upper and lower quartiles (dotted lines) and the median (solid line). **(E)** The ratio of IKKα and IKKβ (IKK) to NEMO copy number for individual cell types at the steady state. Individual replicate values are shown, median value is indicated by solid line, boxes extend from the upper to lower quartile, and whiskers indicate minimum and maximum values. CD4, CD4^+^ T cells; CD4 CM, CD4^+^ central memory T cells; CD4 EM,CD4+ effector memory T cells; CD4 EMRA, CD4^+^CD45^+^ effector memory T cells; CD8, CD8^+^ T cells; T reg, regulatory T cells; NK bright, natural killer cells CD56^high^; NK dim, natural killer cells CD56^low^; MO classical, monocytes CD14^+^CD16^−^; MO intermediate, monocytes CD14^+^CD16^low^; MO non-classical, monocytes CD14^+^CD16^+^; pDCs, plasmacytoid dendritic cells.

The IKK complex is a tripartite complex composed of the homologous kinases IKKα and IKKβ, and the scaffold protein NEMO [15], [16] (Fig. 2A). Although the IKK complex is essential for the activation of NF-ĸB by most stimuli, the stoichiometry of IKKα, IKKβ and NEMO remains to be conclusively defined. A ratio of IKKα_1_:IKKβ_1_:NEMO_2_ has been recently proposed based on crystallographic and quantitative analyses of the interaction between N-terminal NEMO and C-terminal IKK fragments [17] and reconstitution of the IKK complex in yeast [18]. Here, analysis of IKK component copy numbers across all immune cell types reveals that IKKβ is the most abundant component of the IKK complex, followed by NEMO and IKKα, which is a relatively minor component by abundance (Fig. 2B). This pattern of relative abundance is typified in CD4^+^ T cells, naïve B cells, monocytes and myeloid dendritic cells (mDCs) (Fig. 2C). Calculating the ratio of IKKα/β:NEMO across all cell types indicates that the overall average ratio of IKKs to NEMO is 2:1 (Fig. 2D). Assuming all NEMO is bound to IKK proteins these data suggest that the most likely stoichiometry of the IKK complex is (IKKα/β)_2_:NEMO_1_. Alternatively, the proposed (IKKα/β)_2_:NEMO_2_ configuration would require the presence of significant pools of IKKs unbound by NEMO. This analysis also reveals that the majority of IKK complexes are composed of IKKβ homodimers, assuming all IKKs are dimerised. In addition, there is significant variation in IKKα/β:NEMO ratios across cell types which suggests that the composition of the IKK complex may vary in different cells (Fig. 2E). For example, the relative abundancies of the IKK components in CD4^+^ T cells (IKKα/β:NEMO 2.87 ± 0.32) would allow an IKK complex composed entirely of IKKβ and NEMO alongside free IKKα. In contrast, neutrophils appear to have by far the lowest ratio of IKKα/β:NEMO (0.54 ± 0.26) suggesting that there are significant amounts of NEMO not bound to IKKs in these cells. Much of the previous work on the composition of the IKK complex has come from studies involving cell lines such as HeLa [5]. Analysis of available quantitative proteomic data from HeLa cells [12], [13] shows that these cells have a strikingly different profile of IKKα, IKKβ and NEMO abundances unlike any of the immune cell types analysed here. These data show that the levels of IKKα and IKKβ in HeLa cells are equivalent to one another and are 2–3 times lower than those of NEMO (Supplementary Fig. 1). What influence different abundancies of IKK complex constituents may have on the stoichiometry and function of the IKK complex however, is not clear.

### 3.2 The IĸB family of proteins show cell type specific patterns of abundance

**Fig. 3:**
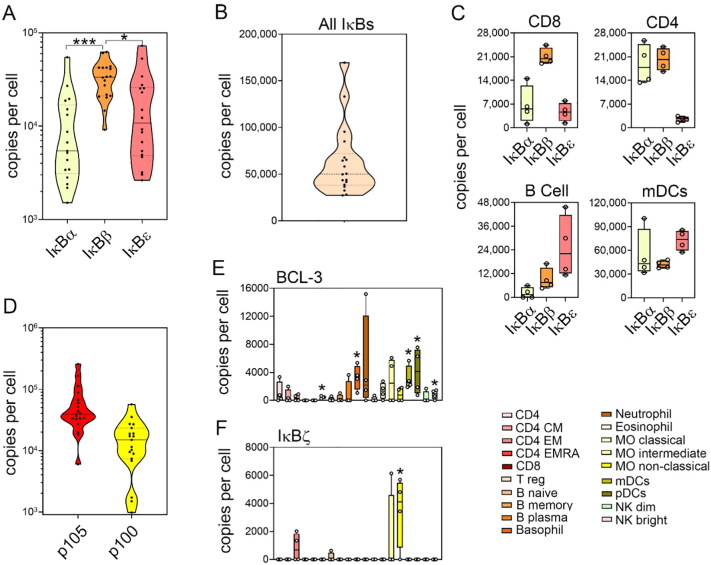
**Cell type specific patterns of IĸB protein levels at the steady state. (A)** Distribution of IĸBα, IĸBβ and IĸBε protein abundance across all cell types at the steady state. Mean values of replicate samples for each cell type are plotted. Violin plot shows upper and lower quartiles (dotted lines) and the median (solid line). **(B)** Combined copy numbers of IĸBα, IĸBβ and IĸBε for all cell types at the steady state. Violin plots shows mean value for each cell type, upper and lower quartiles (dotted lines) and the median (solid line). **(C)** Copy number per cell at the steady state of IKKα, IKKβ and NEMO for CD4^+^ T cells, CD8^+^ T cells, naïve B cells, and myeloid DCs (mDCs). Individual replicate values are shown, mean value is indicated by solid line, boxes extend from the upper to lower quartile and whiskers indicate minimum and maximum values. **(D)** Distribution of p105 and p100 estimated protein abundance across all cell types at the steady state. Mean values of replicate samples for each cell type are plotted. Violin plot shows upper and lower quartiles (dotted lines) and the median (solid line). **(E)** Copy number per cell of BCL-3 and **(F)** IĸBζ for individual cell types. For (E) and (F) individual replicate values are shown, median value is indicated by solid line, boxes extend from the upper to lower quartile and whiskers indicate minimum and maximum values. Cell types where protein is present (*p* < 0.05) are indicated by *. CD4, CD4^+^ T cells; CD4 CM, CD4^+^ central memory T cells; CD4 EM, CD4+ effector memory T cells; CD4 EMRA, CD4^+^CD45^+^ effector memory T cells; CD8, CD8^+^ T cells; T reg, regulatory T cells; NK bright, natural killer cells CD56^high^; NK dim, natural killer cells CD56^low^; MO classical, monocytes CD14^+^CD16^−^; MO intermediate, monocytes CD14^+^CD16^low^; MO non-classical, monocytes CD14^+^CD16^+^; mDC, myeloid dendritic cells; pDCs, plasmacytoid dendritic cells.

IĸBα, IĸBβ and IĸBε are the proto-typical IĸB proteins that sequester NF-ĸB dimers in the cytoplasm of resting cells and undergo signal-induced phosphorylation, degradation and re-synthesis to control NF-ĸB transcriptional activity [4]. There is a wide distribution of IĸBα, IĸBβ and IĸBε protein levels across all cell types (Fig. 3A), however the total number of IĸB proteins per cell is less variable (Fig. 3B). In the majority of cells, including CD8^+^ T cells, IĸBβ is the most highly expressed IĸB protein (Fig. 3A and Supplementary Fig. 2) (Fig. 3C). However, there are distinct patterns of IĸB protein abundancies found in different cell types. For example, CD4^+^ T cells express equivalent levels of IĸBα and IĸBβ that are significantly higher than that of IĸBε, while IĸBε is by far the pre-dominant IĸB protein in naïve B cells which express relatively low levels of IĸBα. In myeloid DCs IĸBε is also the pre-dominant IĸB protein, while monocytes express equivalent levels of all three IĸB proteins (Fig. 3C). The potential importance of different IĸB proteins in different cells is not clear, but the patterns identified here suggest cell specific roles for individual IĸB proteins.

The NF-ĸB precursor proteins p105 and p100 also function as cytoplasmic IĸB proteins by binding NF-ĸB subunits through their C terminal ankyrin repeat domains [4]. The exact levels of precursor proteins and processed subunits cannot be directly determined from the available proteomic data since this data comprises peptides originating from the processed subunits p50 and p52 as well as p105 and p100. However, assuming that all NF-ĸB dimers are bound to a single IĸB and are thereby inactive in the steady state, we calculated the levels of p105 and p100 using the protein levels of the NF-ĸB subunits (see next section). This suggests that p105 is the most abundant IĸB protein in all cell types, with an average abundance greater than that of the other IĸBs combined, while on average p100 levels are closer to those of IĸBε (Fig. 3D).

The atypical IĸB proteins BCL-3, IĸBζ, IĸBNS and IĸBη have been reported previously to be expressed at low levels in resting cells. The proteomic data analysed here supports these findings and shows that the expression levels of these factors in most cells is below the limit of detection. Significant levels of IĸBNS or IĸBη above the detection threshold were not found in any of the cells types analysed (data not shown) while BCL-3 protein is detected in T regulatory cells, basophils, myeloid and plasmacytoid dendritic cells and NK cells (Fig. 3E). IĸBζ was expression was detected in CD4^+^ effector memory cells, naïve B cells, and intermediate and non-classical monocytes (Fig. 3F). This indicates that, at least in resting cells, the atypical proteins may have non-redundant, cell type specific roles. Of course, it is to be noted that levels of proteins below the threshold for detection does not mean these factors are absent but it does re-enforce that these factors are present in very low levels in resting cells.

### 3.3 The abundance of the NF-ĸB subunits RelB and cRel varies widely across cell types

**Fig. 4:**
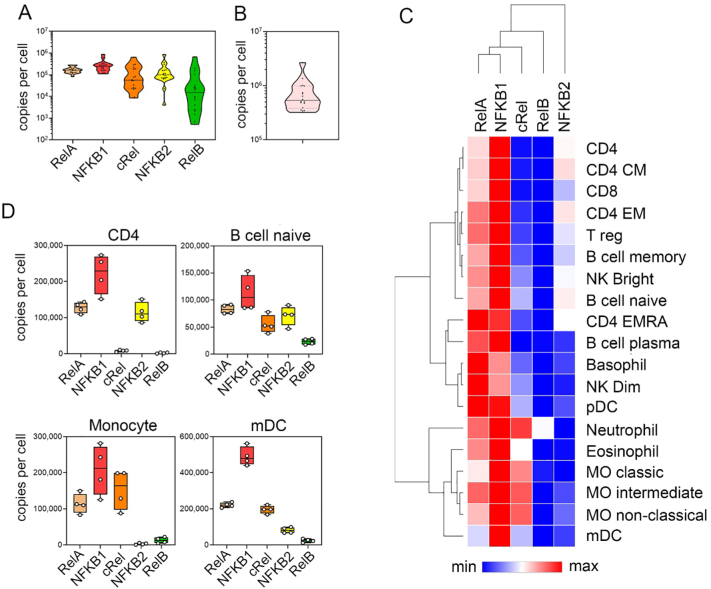
**Abundancies of NF-ĸB subunits in different cell types. (A)** Distribution of RelA, NFKB1, cRel, NFKB2 and RelB protein abundance across all cell types at the steady state. Mean values of replicate samples for each cell type are plotted. Violin plot shows upper and lower quartiles (dotted lines) and the median (solid line). **(B)** Combined copy numbers of all subunits for all cell types at the steady state. Violin plots shows mean value for each cell type, upper and lower quartiles (dotted lines) and the median (solid line). **(C)** Hierarchical clustering of the relative levels of NF-ĸB subunits in individual cell types at steady state using the mean value of replicate samples for each cell type. Heat map presents relative minimum and maximum levels per row. **(D)** Copy number per cell at the steady state of each subunit for CD4 T cells, naïve B cells, classical monocytes and myeloid DCs (mDC). Individual replicate values are shown, mean value is indicated by solid line, boxes extend from the upper to lower quartile and whiskers indicate minimum and maximum values. CD4, CD4^+^ T cells; CD4 CM, CD4^+^ central memory T cells; CD4 EM, CD4+ effector memory T cells; CD4 EMRA, CD4^+^CD45^+^ effector memory T cells; CD8, CD8^+^ T cells; T reg, regulatory T cells; NK bright, natural killer cells CD56^high^; NK dim, natural killer cells CD56^low^; MO classical, monocytes CD14^+^CD16^−^; MO intermediate, monocytes CD14^+^CD16^low^; MO non-classical, monocytes CD14^+^CD16^+^; mDC, myeloid dendritic cells; pDCs, plasmacytoid dendritic cells.

The five subunits of the NF-ĸB family are generally considered to be ubiquitously expressed. Analysis of immune cell data supports this and shows that all five NF-ĸB subunits are detected in all cell types (Fig. 4A). The average total number of NF-ĸB subunits per cell ranged from 1.2 × 10^6^ in neutrophils to 3.4 × 10^5^ in naïve B cells with an average of 5.9 × 10^5^ per cell across all cell types (Fig. 4B). The relative protein levels of each subunit varies within a given cell type, however NFKB1 (p105/p50) is the most abundant NF-ĸB subunit in most cell types at the steady state (Fig. 4A and C). RelA, NFKB1 and NFKB2 (p100/p52) show relatively low variation in abundance between different cell types in contrast to RelB and cRel which vary widely in abundance across cell types. RelB is the least abundant subunit in all cell types at the steady state with the exception of neutrophils (Fig. 4C) There appears to be an inverse relationship between the levels of cRel and NFKB2 protein (Fig. 4C); for example, the relative abundance of NFKB2 protein is highest in T lymphocytes such as CD4^+^ T cells where relative levels of cRel protein are low (Fig. 4C and D), while the highest relative abundance of cRel is found in myeloid cells such as monocytes where the relative abundance of NFKB2 is low (Fig. 4C and D).

### 3.4 A network model of the NF-ĸB pathway in steady state and activated cells

**Fig. 5:**
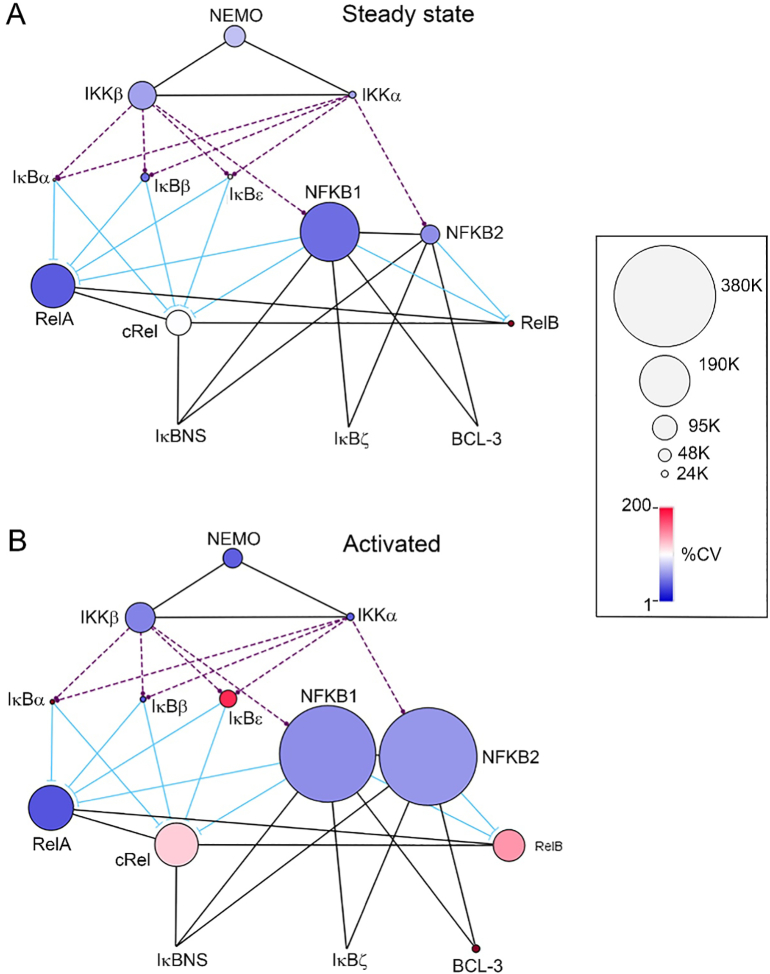
**NF-ĸB pathway network in steady state and activated cells.** Mean copy number per cell of each factor from replicate samples for each cell type were used to calculate mean values for each factor in the network in the (**A**) steady state and (**B**) activated state.. The size of each node is directly proportional to the mean protein abundance. The node colour reflects the calculated coefficient of variation (%CV) of each factor. Interactions are indicated by black lines; phosphorylation by dashed purple lines; and inhibition by blue lines. (For interpretation of the references to colour in this figure legend, the reader is referred to the web version of this article.)

We next constructed a network model of the NF-ĸB pathway using the average abundance of each component across all cell types. This revealed a remarkable conservation of stoichiometry of NF-ĸB pathway components across different cell types with relatively low variation in copy number per cell between different cell types at the steady state for most components (Fig. 5A). The factors with the most variable expression levels between cell types are IĸBα, IĸBε, RelB and cRel (Fig. 5A, Fig. 3A and Fig. 4A). This model also highlights the low levels of IĸB proteins (IĸBα, IĸBβ and IĸBε) relative to NF-ĸB subunits, further indicating that the p105 protein is the major IĸB protein. The conserved NF-ĸB pathway structure in primary human immune cells is distinct from that observed in cell lines such as HeLa, HEK293, Jurkat and U2OS using similar quantitative proteomic data [12], [13] (Supplementary Figs. 3–6). This is perhaps unsurprising considering the transformed nature of these cell lines, but it may have implications for the relevance of data from these cell systems to primary immune cells. We also utilised a recent study of mouse CD4^+^ and CD8^+^ T cells [14] to compare NF-κB networks between mouse and human T cells. This comparison reveals that overall the structure of the NF-κB pathway is highly similar between mouse and human cells, in particular the stoichiometry of the IKK complex components, and the relative abundancies of IκBα, IκBβ and IκBε (Supplementary Fig. 7). There is however a clear difference in the relative abundancies of RelA and NFKB1 between mouse and human cells. In mouse CD4^+^ and CD8^+^ cells RelA is the most abundant component of the NF-κB pathway and is 2–3 fold more abundant than NFKB1, while in human CD4^+^ and CD8^+^ T cells NFKB1 is the most abundant component and is approximately 2 fold more abundant than RelA.

**Fig. 6:**
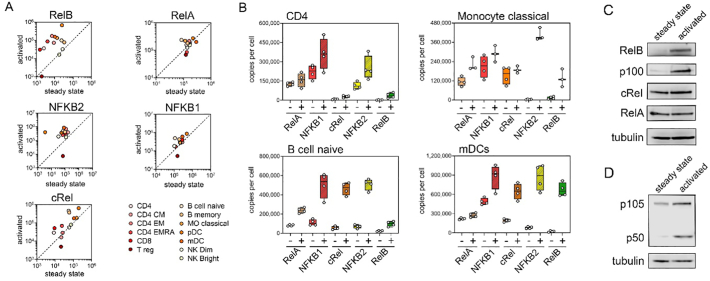
**Activation induces changes in NF-ĸB subunit levels. (A)** NF-ĸB subunit levels in steady state versus activated cells. Scatter plot shows mean copy number of replicate samples for steady state versus activated state for each cell type. **(B)** Copy number per cell of each subunit at the steady state (−) and activated state (+) for CD4 T cells, naïve B cells, classical monocytes and myeloid DCs (mDC). Individual replicate values are shown, mean value is indicated by solid line, boxes extend from the upper to lower quartile and whiskers indicate minimum and maximum values. **(C)** Western blot analysis of NF-κB subunits in primary human monocytes at the steady state and following activation (LPS treatment). Cells were lysed and western blot performed using the antibodies indicated. Tubulin expression was used as a loading control. CD4, CD4^+^ T cells; CD4 CM, CD4^+^ central memory T cells; CD4 EM, CD4+ effector memory T cells; CD4 EMRA, CD4^+^CD45^+^ effector memory T cells; CD8, CD8^+^ T cells; T reg, regulatory T cells; NK bright, natural killer cells CD56^high^; NK dim, natural killer cells CD56^low^; MO classical, monocytes CD14^+^CD16^−^; MO intermediate, monocytes CD14^+^CD16^low^; MO non-classical, monocytes CD14^+^CD16^+^; mDC, myeloid dendritic cells; pDCs, plasmacytoid dendritic cells.

We next generated a network model using data from activated cells (CD4, CD4 CM, CD4 EM, CD4 EMRA, CD8, T reg, naïve B cells, memory B cell, monocytes, pDC, mDC and NK cells). This revealed that across all cell types the abundances of the IKK complex, RelA and IĸBβ are not substantially altered following activation (Fig. 5B). However, there is a remarkable activation-induced increase in the protein levels of NFKB2 and RelB that is consistent across different cell types (Fig. 5B and 6A). There is also an increase in p105 protein expression in most activated cell types while increases in cRel and IĸBε protein levels occur in specific cell types following activation (Fig. 5B and 6A). Most cells types show significant increases in RelB after activation, with the exception of NK and T reg cells (Fig. 6A). Monocytes show the greatest increase in NFKB2 levels following activation with approximately 110 fold more NFKB2 protein per cell compared to the steady state, while in the same cells RelB protein levels are increased by 10 fold (Fig. 6B). Activated CD4^+^ T cells show a robust 14 fold increase in RelB protein levels compared to steady state cells, and a more modest 2 fold increase in NFKB2 protein relative to the steady state (Fig. 6B). As expected [19], activated CD4^+^ T cells have increased cRel protein levels compared to steady state cells. The approximately 3 fold increase in cRel protein in CD4^+^ T cells is similar to that seen in mDCs but lower than the 8 fold increase of cRel in activated naïve B cells (Fig. 6B). We confirmed the patterns of NF-κB subunits expression in steady state and LPS stimulated primary monocytes using western blot approaches (Fig. 6C and D). Overall, this analysis reveals that the NF-ĸB pathway in activated cells is distinct from the steady state and that differences occur in a cell type specific manner.

### 3.5 Distinct profiles of IĸB proteins in activated cells

**Fig. 7:**
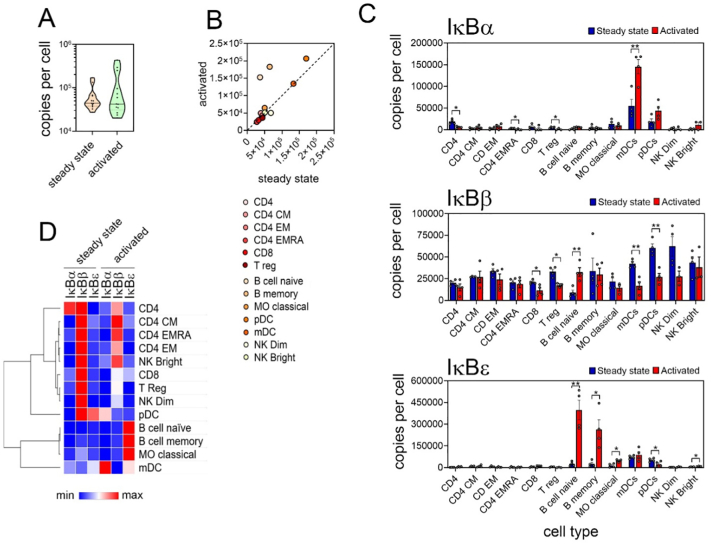
**Cell type specific changes in IĸB protein abundance following activation. (A)** Copy number per cell of IĸBα, IĸBβ and IĸBε for each cell type in the steady state and activated state. Graphs show the mean of individual replicate samples +/− SEM. **(B)** Combined copy number per cell of IĸBα, IĸBβ and IĸBε for cells in the steady state and activated state. Violin plots show mean values of replicate samples for each cell type, the median (solid line) and the upper and lower quartiles (dotted line). **(C)** Combined copy number per cell of IĸBα, IĸBβ and IĸBε in steady state versus activated cells. Scatter plot shows mean copy number of replicate samples for steady state versus activated state for each cell type. * *p* < 0.05, ** *p* < 0.003. **(D)** Hierarchical clustering of the relative levels of IĸB proteins in individual cell types at steady state and activated state using the mean value of replicate samples for each cell type. Heat map presents relative minimum and maximum levels per row. CD4, CD4^+^ T cells; CD4 CM, CD4^+^ central memory T cells; CD4 EM, CD4+ effector memory T cells; CD4 EMRA, CD4^+^CD45^+^ effector memory T cells; CD8, CD8^+^ T cells; T reg, regulatory T cells; NK bright, natural killer cells CD56^high^; NK dim, natural killer cells CD56^low^; MO classical, monocytes CD14^+^CD16^−^; MO intermediate, monocytes CD14^+^CD16^low^; MO non-classical, monocytes CD14^+^CD16^+^; mDC, myeloid dendritic cells; pDCs, plasmacytoid dendritic cells.

The activation induced degradation and re-synthesis of IĸB proteins is a critical regulatory feature of the NF-ĸB pathway [4]. Analysis of IĸBα, IĸBβ and IĸBε protein levels across all cell types in the resting and activated states reveals that the total copy number per cell of typical IĸB proteins is not significantly altered following activation (Fig. 7A). This also holds true for individual cell types with the exception of naïve and memory B cells, which demonstrate increased levels of IĸB proteins following activation (Fig. 7B). Of note, it is pre-dominantly the increased expression of IĸBε in activated B cells that accounts for the observed increase in IĸBε in the NF-ĸB network model of activated cells above (Fig. 5B). While the total number of IĸB proteins remains the same in activated cells relative to the steady state, activation appear to alter the relative abundancies of each IĸB protein in some cells. For example, mDCs and pDCs have reduced levels of IĸBβ but increased levels of IĸBα following activation (Fig. 7C and D).

**Fig. 8:**
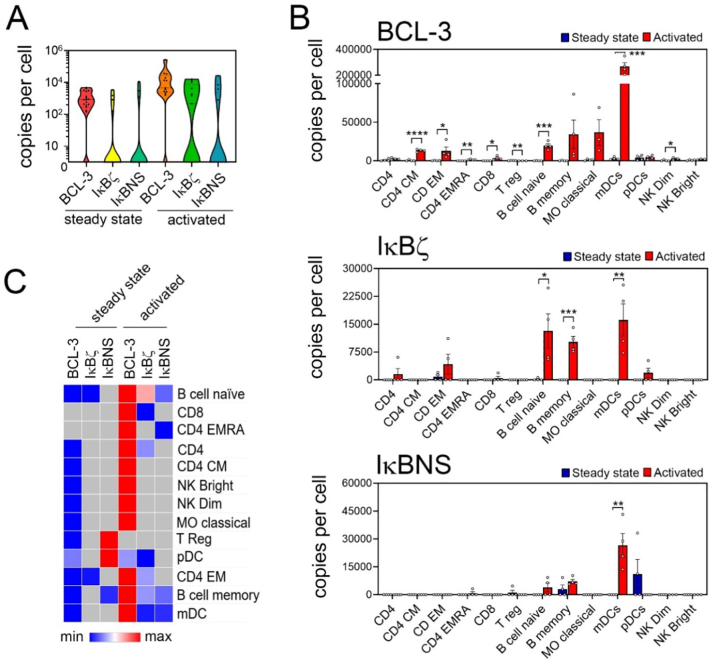
**Cell type specific changes in atypical IĸB protein abundance following activation. (A)** Combined copy numbers per cell of IĸBα, IĸBβ and IĸBε for cells in the steady and activated state. Violin plots show mean values of replicate samples for each cell type, the median (solid line) and the upper and lower quartiles (dotted line). Only data from cell types expressing at least one protein in at least one state is shown. **(B)** Copy number per cell of BCL-3, IĸBζ and IĸBNS for each cell type in the steady state and activated state. Graphs show the mean of individual replicate samples +/− SEM. * *p* < 0.05, ** *p* < 0.003, *** *p* < 0.001. **(C)** Hierarchical clustering of the relative levels of IĸB proteins in individual cell types at steady state and activated state using the mean value of replicate samples for each cell type. Heat map presents relative minimum and maximum levels per row. Grey colour indicates that no expression was detected. CD4, CD4^+^ T cells; CD4 CM, CD4^+^ central memory T cells; CD4 EM,CD4+ effector memory T cells; CD4 EMRA, CD4^+^CD45^+^ effector memory T cells; CD8, CD8^+^ T cells; T reg, regulatory T cells; NK bright, natural killer cells CD56^high^; NK dim, natural killer cells CD56^low^; MO classical, monocytes CD14^+^CD16^−^; MO intermediate, monocytes CD14^+^CD16^low^; MO non-classical, monocytes CD14^+^CD16^+^; mDC, myeloid dendritic cells; pDCs, plasmacytoid dendritic cells.

As previously noted, the atypical IĸB proteins BCL-3, IĸBζ and IĸBNS are absent or expressed at very low levels in cells at the steady state [4] (Fig. 3). Activation increases the expression of these proteins in a cell type specific manner(Fig. 8A). BCL-3 is the most abundant atypical IĸB protein in both the steady state (Fig. 3E) and activated cells, with a particularly high expression levels in activated mDCs (Fig. 8B). Of note, activation of mDCs also induces a significant increase in IĸBζ and IĸBNS protein levels, suggesting that the atypical IĸB proteins may play an important role in regulating NF-ĸB activity these cells (Fig. 8B). IĸBNS is not observed in activated cells other than mDCs while IĸBζ protein is also significantly increased in activated memory and naïve B cells. IĸBη is not detected in any cells following activation (data not shown). These analyses indicate that individual atypical IĸB proteins may have cell type specific roles following activation.

**Fig. 9:**
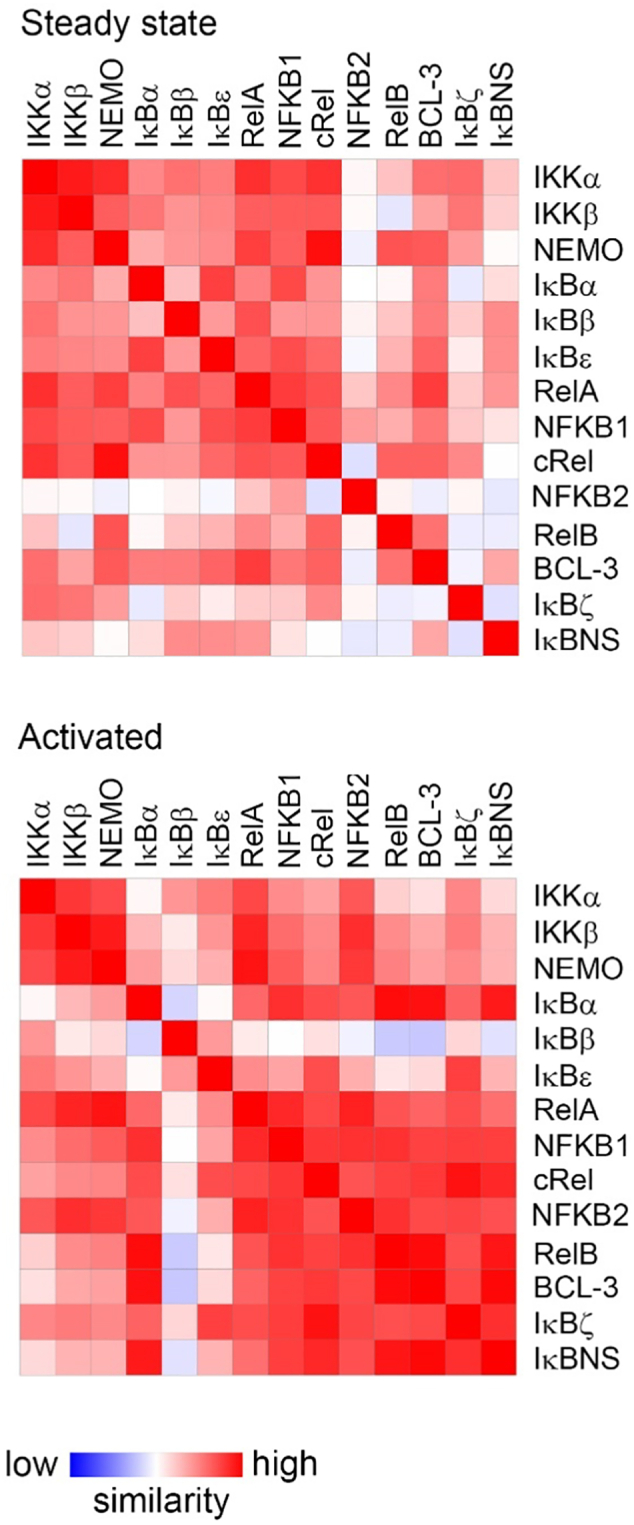
**Distinct NF-ĸB architecture in steady stated and activated cells.** Mean copy numbers of each factor per cell for all cell types were used to generate a similarity matrix based on relative abundances. Cells at the steady state have similar patterns of expression for IKKα, IKKβ, NEMO, the IĸB proteins α, β and ε, and the NF-ĸB subunits RelA, p105 and cRel. Following activation cells share similar patterns of expression of all NF-ĸB subunits and the atypical IĸB proteins BCL-3, IĸBζ and IĸBNS.

To provide a further comparison of the overall structure of the NF-ĸB pathway in resting and activated cells we next generated a similarity matrix based on average protein copy number per cell of all factors across all cell types. This analysis revealed that the core NF-ĸB components in the steady state are the IKK complex, IĸBα, IĸBβ, IĸBε, RelA, NFKB1 and cRel (Fig. 9), essentially comprising the classical NF-κB pathway. However, in activated cells the atypical IĸB proteins BCL-3, IĸBζ and IĸBNS replace IĸBα, IĸBβ and IĸBε in the similarity matrix (Fig. 9). This further supports the concept that the atypical IĸBs are important modifiers of NF-ĸB activity following activation. In addition, the RelB and NFKB2 subunits join the other subunits as part of the core NF-κB pathway in activated cells, reflecting the increased levels of these factors in activated cells.

## 4 Discussion

Quantitative proteomic data offer a unique opportunity to assess the current model of the NF-ĸB pathway. In this study we have used open access data from primary human immune cells in the steady and activated states to measure the abundance and stoichiometry of the core components of the pathway. Overall the analysis shows a strong conservation of pathway structure across different cell types and lineages. The analysis also reveals that the structure of the NF-ĸB pathway is dynamic in response to activating stimuli and that increased RelB and NFKB2 protein levels is a general feature of activated immune cells. The majority of the core pathway components are present in highly similar abundancies in different cell types, revealing that the NF-ĸB pathway in different cell types is broadly the same in terms of structure and abundance. An exception to this is the low abundance of NF-κB pathway components in T regulatory cells, an observation which correlates strongly with the requirement for these cells to supress NF-κB activity to maintain function [20].

While the broad similarity of the NF-κB structure in primary immune cells will allow for the extrapolation of observations in one cell type to another, the obvious dissimilarity to cells commonly used to study NF-ĸB suggests caution in extrapolating findings from cell lines to primary cells. This is clearly illustrated when considering the stoichiometry of the IKK complex which is widely accepted to be composed of equal amounts of IKKα and IKKβ, with a number of different stoichiometries proposed including IKKα_1_:IKKβ_1_:NEMO_2_. However, assuming that there is no free pool of these proteins outside the IKK complex, the analysis here indicates a stoichiometry of (IKKα/β)_2_:NEMO_1_. Moreover, in contrast to cell lines such as HeLa, the relative excess of IKKβ over IKKα in primary cells indicates that the majority of IKK complexes are likely composed of IKKβ homodimers. Indeed, in some cell types such as CD4^+^ T cells, the excess of IKKβ over IKKα suggests the possibility that the IKK complex is composed exclusively of IKKβ and NEMO, since NEMO does not directly bind IKKα [5]. It is not known whether the functional properties of IKK complexes composed of IKKβ homodimers are different to those composed of IKKα and IKKβ heterodimers but it is worthy of investigation.

The non-canonical pathway of NF-ĸB activation has been predominantly linked to a relatively small number of TNFR superfamily members including CD40, RANK, Lymphotoxin β receptor and BAFF receptor [6]. It requires IKKα, but not IKKβ nor NEMO, and involves the activation of NF-ĸB dimers composed of RelB and p52 [7]. From the analysis here we see that RelB is the least abundant subunit in almost all cell types at the steady state indicating a potentially limited role for the non-canonical pathway. However, in activated cells there is a significant increase in the abundance of RelB and NFKB2 in most cells, indicating that the non-canonical pathway may play an important role in immune cells post activation and may indeed be a general feature of activated immune cells, independent of the activating stimulus. The established role of the non-canonical pathway in controlling B cell homeostasis in the periphery [21] and T cell memory [22] suggests that this pathway may be broadly important in deciding the cell fate of most immune cells following stimulation.

IĸBα is the prototypic member of the IĸB family of proteins and its deletion in mice results in death shortly after birth [23]. However, the analysis here reveals that IĸBβ is more abundant than IκBα in the majority of cells at the steady state with IĸBε predominant in naïve B cells and mDCs. The roles of IĸBβ and IĸBε in regulating the NF-ĸB are less well understood although the available data suggests that they have distinct properties to IĸBα. IĸBβ plays a role in promoting the duration of NF-ĸB transcriptional activity on certain genes such as IL-1β [24], [25] and may also be important in the generation of RelA homodimers [26], while the delayed kinetics of IKK induced degradation of IĸBε may play a role in shaping the duration of NF-ĸB activity [27], [28]. Understanding the biological roles of IĸB proteins is complicated by functional redundancy in vivo [29] and the importance of feedback loops in the NF-ĸB pathway in setting protein levels [30]. However, the significant amounts of IĸBβ and IĸBε in primary immune cells suggests that the contribution of these factors to the NF-ĸB pathway may not be fully appreciated. The low abundancies of IκBα, β and ε proteins relative to RelA are surprising, as is the relatively low abundance of IκBα in most cells, particularly when considering the key role played by IκBα in regulating NF-κB activity. Conversely, NFKB1 constitutes the most abundant component of the NF-κB pathway but *Nfkb1* deletion in mice leads to a relatively moderate immune phenotype when compared to deletion of *Nfkbia* [3]. How IκBα achieves it's regulatory power over NF-κB activity while present at such relatively low abundance is unclear. Analysis of a recent dataset generated from mouse CD4^+^ and CD8^+^ T cells [14] reveals that the structure of the NF-κB pathway is highly similar between mouse and human for these cell types with one notable exception. In mouse CD4^+^ and CD8^+^ cells RelA is the most abundant component of the NF-κB pathway while in human CD4^+^ and CD8^+^ T cells NFKB1 is the most abundant component. It is noteworthy that *Nfkb1*^+\-^ mice do not have an overt immune phenotype [31] while *NFKB1* haploinsufficiency is the most common monogenic cause of combined variable immunodeficiency in Europeans [32]. Thus, it is tempting to speculate that the greater relative abundance of NFKB1 in human cells may underlie its greater importance in humans compared to mouse.

The atypical IĸB proteins BCL-3, IĸBζ, IĸBNS and IĸBη are located predominantly in the nucleus where they act to modulate the activity of specific NF-ĸB dimers [3]. Previous reports that these proteins are mostly expressed a low levels at the steady state but induced following activation are reflected in the analysis presented here [33], [34], [35], [36]. IĸBη was not detected in any of the data analysed here indicting that it may not be a significant regulator of NF-ĸB in immune cells. As with the typical IĸB proteins, the expression profiles of atypical IĸBs are cell type specific with BCL-3 being the most abundant and most widely expressed. The functional impact of these different profiles on NF-ĸB activity is unclear and additional experimental investigation will be needed to provide greater understanding.

An overview of the patterns of abundance between the components of the NF-ĸB pathway in all cell types identifies the core pathway components in both the steady and activated states. Unsurprisingly, the IKK complex, the canonical subunits RelA, NFKB1 and cRel, and the IĸB members IĸBα, IĸBβ and IĸBε form the core in the steady state. In the activated state the core components of the pathway are different and includes all the NF-ĸB subunits including NFKB2 and RelB and the atypical IĸB proteins BCL-3, IĸBζ and IĸBNS, reflecting changes in the abundance of RelB, NFKB2 and the atypical IĸB proteins. This analysis raises important questions when considering how the NF-ĸB pathway may be exploited for therapeutic benefit. To date, the key strategy for preventing NF-ĸB dependent inflammation has been to inhibit the IKK complex using IKKβ kinase inhibitors, which unfortunately leads to severe toxicity that precludes their clinical application [37]. However, if we consider that activated cells, rather than cells in the steady state, are the key contributors to inflammatory disease then the differences in the architecture of the NF-ĸB pathway between these two states may point towards alternative strategies. The almost universal increase in RelB and NFKB2 abundance in immune cells activated by diverse stimuli suggests that the non-canonical pathway may be a more effective target. The recent development of IKKα specific kinase inhibitors may allow for the selective modulation of activated cells over those in the steady state [38]. Such an approach necessitates a clear understanding of the role of the non-canonical pathway in activated immune cells and the consequences of its blockade. Similarly, the expression of atypical IĸB proteins in activated cells in cell type specific patterns identifies these as factors that distinguish between immune cells in the activated and steady states. While these factors make difficult drug targets, proof of principle experiments have shown that BCL-3 mimetic peptides are effective at inhibiting pro-inflammatory cytokine production in vitro and in vivo [39].

In summary, quantitative proteomic analysis offers unique insights into the structure of the NF-ĸB pathway in primary cells which differs in important ways from those of cell lines commonly used in its study. It raises important questions on the nature of the IKK complex and the role of individual IĸB proteins that require further experimental investigation in order to answer. Moreover, it shows that the structure of the NF-ĸB pathway is dynamic following cellular activation suggesting new approaches to harnessing this pathway for therapeutic benefit.

## Declaration of Competing Interest

The authors have no conflict of interest.

## Appendix A Supplementary data

Supplementary material Image 1
