# Supplementary material for: Defining the structure of the NF-ĸB pathway in human immune cells using quantitative proteomic data
Source: Cell Signal. 2021 Dec;88:110154. doi: 10.1016/j.cellsig.2021.110154 (PMC8573605; doi:10.1016/j.cellsig.2021.110154)
Supplement: Supplementary file 1 — Supplementary material [file mmc1.docx]

–Supplemental Information

**A**


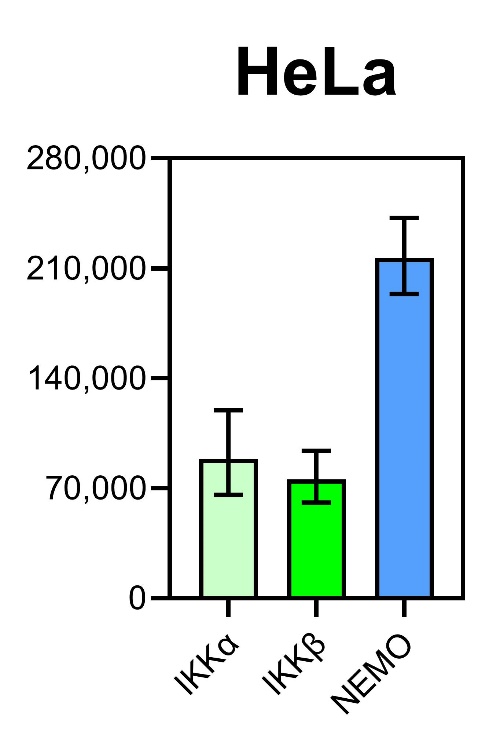


**B**


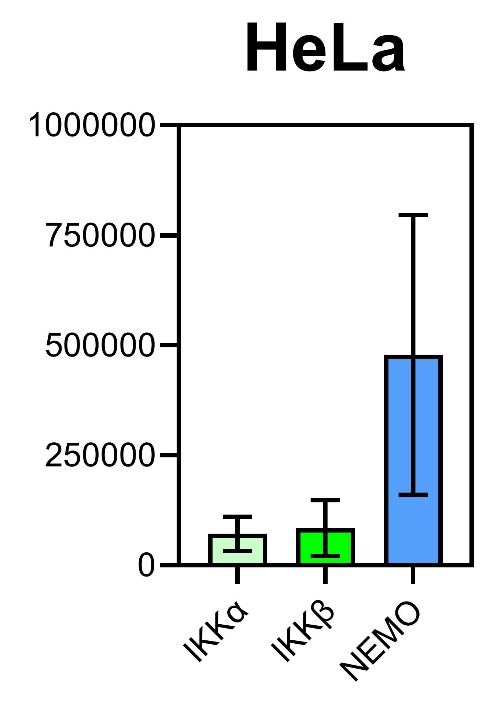


**Supplemental figure 1**. Copies per HeLa cell of IKKα, IKKβ and NEMO from (**A**) Itchak et al (*1*) and (**B**) Geiger et al(*2*).


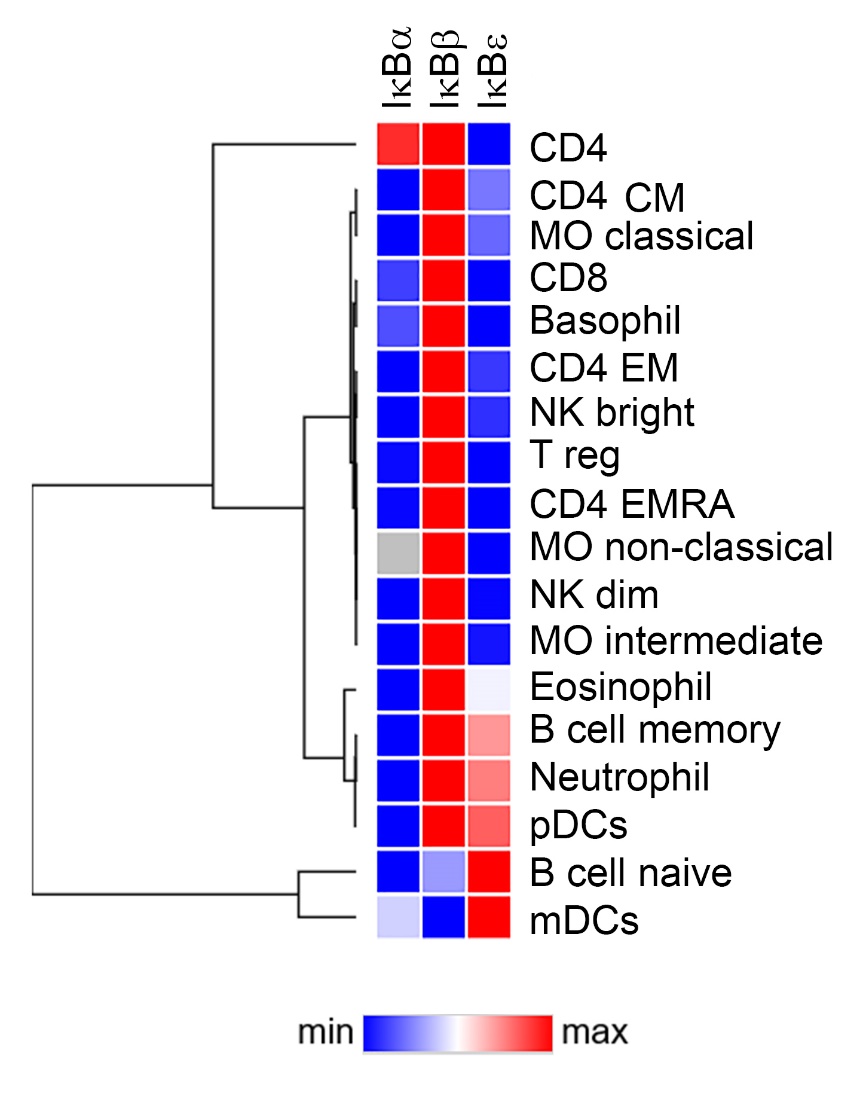


**Supplemental figure 2.** Hierarchical clustering of the relative levels of IĸB proteins in individual cell types at steady state using the mean value of replicate samples for each cell type. Heat map presents relative minimum and maximum copy number per row. CD4, CD4^+^ T cells; CD4 CM, CD4^+^ central memory T cells; CD4 EM,CD4+ effector memory T cells; CD4 EMRA, CD4^+^CD45^+^ effector memory T cells; CD8, CD8^+^ T cells; T reg, regulatory T cells; NK bright, natural killer cells CD56^high^; NK dim, natural killer cells CD56^low^; MO classical, monocytes CD14^+^CD16^-^; MO intermediate, monocytes CD14^+^CD16^low^; MO non-classical, monocytes CD14^+^CD16^+^; mDC, myeloid dendritic cells; pDCs, plasmacytoid dendritic cells.


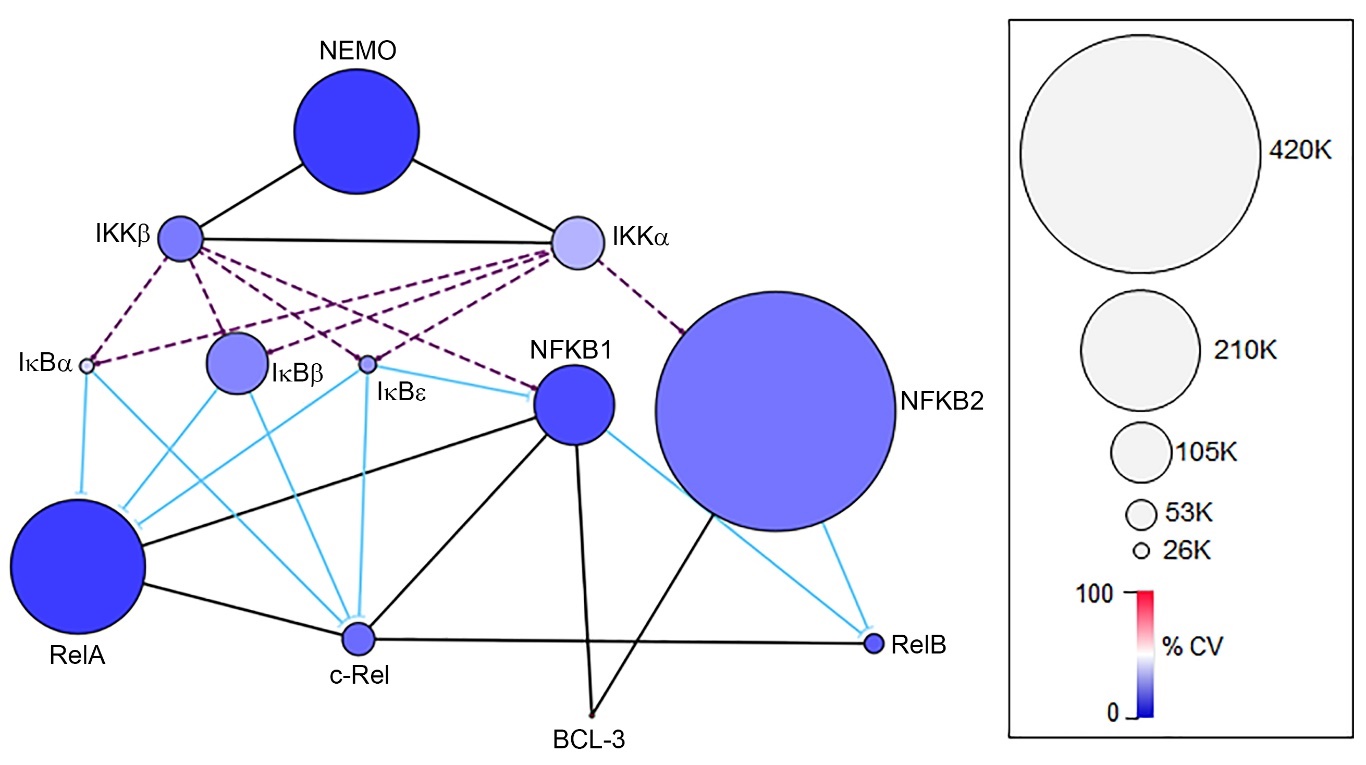


**Supplemental figure 3.** NF-ĸB pathway of HeLa cells generated using quantitative proteomic data from Itzhak et al (*1*). Mean copy number per cell of each factor from replicate samples were used to calculate mean values for each factor in the network. The size of each node is directly proportional to the mean protein abundance as shown in accompanying key. The node colour reflects the calculated coefficient of variation (%CV) of replicate values for each factor as shown in the accompanying key. Interactions are indicated by black lines; phosphorylation by dashed purple lines; and inhibition by blue lines.

**Supplemental figure 4.** NF-ĸB pathway of HEK293 cells generated using quantitative proteomic data from Geiger et al (*2*). Mean copy number per cell of each factor from replicate samples were used to calculate mean values for each factor in the network. The size of each node is directly proportional to the mean protein abundance as shown in accompanying key. The node colour reflects the calculated coefficient of variation (%CV) of replicate values for each factor as shown in the accompanying key. Interactions are indicated by black lines; phosphorylation by dashed purple lines; and inhibition by blue lines.

**Supplemental figure 5.** NF-ĸB pathway of Jurkat cells generated using quantitative proteomic data from Geiger et al (*2*). Mean copy number per cell of each factor from replicate samples were used to calculate mean values for each factor in the network. The size of each node is directly proportional to the mean protein abundance as shown in accompanying key. The node colour reflects the calculated coefficient of variation (%CV) of replicate values for each factor as shown in the accompanying key. Interactions are indicated by black lines; phosphorylation by dashed purple lines; and inhibition by blue lines.

**Supplemental figure 6.** NF-ĸB pathway of U2OS cells generated using quantitative proteomic data from Geiger et al (*2*). Mean copy number per cell of each factor from replicate samples were used to calculate mean values for each factor in the network. The size of each node is directly proportional to the mean protein abundance as shown in accompanying key. The node colour reflects the calculated coefficient of variation (%CV) of replicate values for each factor as shown in the accompanying key. Interactions are indicated by black lines; phosphorylation by dashed purple lines; and inhibition by blue lines.

**Supplemental figure 7.** NF-ĸB pathway of **(A)** mouse and **(B)** human CD4 and CD8 T cells. generated using quantitative proteomic data from Marchingo *et al* (*3*) and Rieckmann *et al* (*4*) respectively. Mean copy number per cell of each factor from replicate samples were used to calculate mean values for each factor in the network. The size of each node is directly proportional to the mean protein abundance as shown in accompanying key. The node colour reflects the calculated coefficient of variation (%CV) of replicate values for each factor as shown in the accompanying key. Interactions are indicated by black lines; phosphorylation by dashed purple lines; and inhibition by blue lines.

**References**:

1. Itzhak, D. N., S. Tyanova, J. Cox, G. H. Borner, Global, quantitative and dynamic mapping of protein subcellular localization. *Elife* **5**, (2016).

2. Geiger, T., A. Wehner, C. Schaab, J. Cox, M. Mann, Comparative proteomic analysis of eleven common cell lines reveals ubiquitous but varying expression of most proteins. *Mol Cell Proteomics* **11**, M111 014050 (2012).

3. Marchingo, J. M., L. V. Sinclair, A. J. Howden, D. A. Cantrell, Quantitative analysis of how Myc controls T cell proteomes and metabolic pathways during T cell activation. *Elife* **9**, (2020).

4. Rieckmann, J. C., R. Geiger, D. Hornburg, T. Wolf, K. Kveler, D. Jarrossay, F. Sallusto, S. S. Shen-Orr, A. Lanzavecchia, M. Mann, F. Meissner, Social network architecture of human immune cells unveiled by quantitative proteomics. *Nat Immunol* **18**, 583-593 (2017).
